# Post-exposure intranasal IFNα suppresses replication and neuroinvasion of Venezuelan Equine Encephalitis virus within olfactory sensory neurons

**DOI:** 10.1186/s12974-023-02960-1

**Published:** 2024-01-17

**Authors:** Matthew D. Cain, N. Rubin Klein, Xiaoping Jiang, Hamid Salimi, Qingping Wu, Mark J. Miller, William B. Klimstra, Robyn S. Klein

**Affiliations:** 1 Center for Neuroimmunology & Neuroinfectious Diseases, Washington University School of Medicine, St. Louis, MO USA; 2 Departments of Medicine, Washington University School of Medicine, St. Louis, MO USA; 3 Departments of Pathology and Immunology, Washington University School of Medicine, St. Louis, MO USA; 4 Departments of Neurosciences, Washington University School of Medicine, St. Louis, MO USA; 5 Department of Immunology and Center for Vaccine Research, University of Pittsburgh, Pittsburgh, PA USA

## Abstract

**Background:**

Venezuelan Equine Encephalitis virus** (**VEEV) may enter the central nervous system (CNS) within olfactory sensory neurons (OSN) that originate in the nasal cavity after intranasal exposure. While it is known that VEEV has evolved several mechanisms to inhibit type I interferon (IFN) signaling within infected cells, whether this inhibits virologic control during neuroinvasion along OSN has not been studied.

**Methods:**

We utilized an established murine model of intranasal infection with VEEV and a repository of scRNAseq data from IFN-treated OSN to assess the cellular targets and IFN signaling responses after VEEV exposure.

**Results:**

We found that immature OSN, which express higher levels of the VEEV receptor LDLRAD3 than mature OSN, are the first cells infected by VEEV. Despite rapid VEEV neuroinvasion after intranasal exposure, olfactory neuroepithelium (ONE) and olfactory bulb (OB) IFN responses, as assessed by evaluation of expression of interferon signaling genes (ISG), are delayed for up to 48 h during VEEV neuroinvasion, representing a potential therapeutic window. Indeed, a single intranasal dose of recombinant IFNα triggers early ISG expression in both the nasal cavity and OB. When administered at the time of or early after infection, IFNα treatment delayed onset of sequelae associated with encephalitis and extended survival by several days. VEEV replication after IFN treatment was also transiently suppressed in the ONE, which inhibited subsequent invasion into the CNS.

**Conclusions:**

Our results demonstrate a critical and promising first evaluation of intranasal IFNα for the treatment of human encephalitic alphavirus exposures.

**Supplementary Information:**

The online version contains supplementary material available at 10.1186/s12974-023-02960-1.

## Introduction

Alphaviruses are members of the Togaviridae family of enveloped single-strand RNA arboviruses transmitted by mosquitoes. The arthritogenic Old World alphaviruses include Chikungunya virus (CHIKV), Sindbis virus (SINV), Semliki Forest virus (SFV) and Ross River virus (RRV). The New World alphaviruses, including Venezuelan, Eastern, and Western equine encephalitis viruses (VEEV, EEEV, WEEV), are characterized by the ability to infect the central nervous system (CNS) leading to meningitis and encephalitis, with acute and chronic neurological sequelae [1]. VEEV–IAB/IC serotypes are linked to human and equine epizootic outbreaks, while VEEV-enzootic cycles occur between rodents and mosquitoes. In addition to natural routes of infection, VEEV, along with EEEV and WEEV, may enter the CNS after intranasal (i.n.) exposure, highlighting the possibility of VEEV weaponization via aerosolization. As there are no approved vaccines for public distribution and no treatments for CNS infection with VEEV, there is a need to understand viral entry and innate immune responses along these routes to develop protective measures.

Studies of murine infections with VEEV-enzootic subtype ZPC-738 show that VEEV can enter the CNS through hematogenous spread across an intact blood–brain barrier (BBB) and via anterograde transport along cranial nerves [2, 3]. Astrocytes are the first infected cell during hematogenous entry, with further dissemination within the CNS via infected neurons [3]. Intranasal (i.n.) exposure leads to infection of the olfactory sensory neurons (OSN) of the nasal cavity neuroepithelium, leading to neuroinvasion along axons that cross the cribiform plate into the olfactory bulbs (OB), which results in widespread CNS infection and lethality. Low Density Lipoprotein Receptor Class A Domain Containing 3 (LDLRAD3), was identified as a receptor for VEEV [4–6]. Global deletion of LDLRAD3 suppresses systemic infection during the peripheral prodrome phase during which peripheral mononuclear cells become infected [4]. While prophylactic administration of LDLRAD3-Fc fusion proteins suppresses peripheral infection and neuroinvasion, this does not suppress all replication within the brain. It is not known whether LDLRAD3 is expressed by neurons within the olfactory routes of invasion.

Type I interferons (IFN) signal via auto- and paracrine activation of JAK/STAT downstream of the IFN receptor (IFNAR), which is necessary to control initial VEEV infection [7]. However, VEEV has evolved several mechanisms to inhibit IFNAR signaling within infected cells, including host transcription and translation shutoff by VEEV capsid and non-structural protein (nsP)2, and nsP inhibition of IFNAR-induced STAT1 activation via mechanisms independent of host shut off [8–12]. While systemic, pre-exposure (> 24 h) administration of exogenous IFN controls aerosolized virulent VEEV infection and enhances survival in mice [13], no studies have examined whether IFN has benefit post-exposure. Overall, the multiple routes of entry into the CNS may require specific treatment strategies that depend on the site of initial infection. Alternative to systemic IFN administration, intranasal IFN treatment may uniquely protect the CNS during aerosolized infection. Intranasally administered IFNβ distributes throughout the CNS along olfactory tracts in rats and non-human primates [14, 15]. This route of administration resulted in higher concentrations of the cytokine in the brain, suggesting that high doses of IFN may additionally protect susceptible neurons distant from initial sites of neuroinvasion. Intranasal administration of IFNα is well-tolerated, making this strategy potentially viable for post-exposure treatment of aerosolized VEEV infection [16].

In this study we demonstrate that VEEV initially targets GAP43+ immature (i)OSN within the olfactory neuroepithelium (ONE). Tropism toward iOSNs correlated with higher LDLRAD3 expression within iOSN versus mature (m)OSN, but no broad deficits in innate immunity, as assessed via scRNAseq, were observed in iOSN that would contribute to their enhanced infectivity over mOSN. Despite rapid VEEV neuroinvasion, host nasal cavity and CNS IFN responses are delayed for up to 48 h during VEEV neuroinvasion, representing a potential therapeutic window. Thus, we evaluated the efficacy of single dose recombinant IFNα administered intranasally at the time of or early after infection (0–3 h post-infection), which was able to trigger ISG expression in both the nasal cavity and OB. IFNα treatment delayed onset of sequelae associated with encephalitis and extended survival by several days. VEEV replication after IFN treatment was also transiently suppressed in the ONE, which inhibited subsequent invasion into the CNS. Together these data identify iOSN that express high levels of LDLRAD3 as the initial target of VEEV, define OSN ISG transcriptomic signatures, and demonstrate the efficacy of intranasal delivery of IFNα to protect sites critical to early VEEV–CNS infection. Our results demonstrate a critical and promising first evaluation of such a treatment strategy for human encephalitic alphavirus infection.

## Materials and methods

### Animals

C57BL/6J mice were purchased from Jackson Laboratories (Bar Harbor, ME). Animals were housed under pathogen-free conditions in Washington University School of Medicine animal facilities. All experiments were performed in compliance with Washington University animal studies guidelines.

### Mouse model of VEEV encephalitis

8–10-week-old male mice were inoculated intranasally (10 µL per nostril) with VEEV strain ZPC-738 or ZPC-738-eGFP (10 or 50 pfu, respectively) under anesthesia. ZPC-738-eGFP was a generous gift of William Klimstra (Pittsburg, PA). ZPC-738-eGFP was generated by subgenomic insertion of GFP as a cleavable element between the capsid and PE2 structural proteins, described previously [17]. Parental ZPC-738 or ZPC-738-eGFP are uniformly lethal at 10 pfu. GFP genomic VEEV modification exhibited slight attenuation, delayed expansion/neuroinvasion during early infection (unpublished data), and extended MST [17]. To account for this, ZPC-738-eGFP studies were performed at 50 pfu. Mice were monitored daily for weight loss and scored daily for encephalitic sequelae. Moribund mice were sacrificed by CO_2_ asphyxiation and recorded as dead the following day. Encephalitic score represents a progressive range of behaviors: (1) hunched, ruffled fur, (2) altered gait, slow movement, (3) not moving but responsive, (4) not moving, poorly responsive but upright, (5) moribund, (6) dead.

### Perfusion–fixation and immunohistochemistry

At various times post-infection, mice were anesthetized followed by extensive cardiac perfusion with PBS and perfusion fixation with 4% paraformaldehyde (PFA) in PBS. Tissue was immersion-fixed for an additional 24 h in 4% PFA. For slice preparations of mouse nasal cavities, skulls were decalcified by multiple exchanges 0.5 M EDTA (pH 7.4) in PBS over 7 days followed by PBS and cryoprotection (two-exchanges of 30% sucrose for at least 48 h) and embedding in OCT (Fisher). 10 μm-thick fixed-frozen sagittal sections were hydrated with PBS and blocked for 1 h in blocking solution, 5% normal donkey serum (Santa Cruz Biotechnology) with 0.1% Triton X-100 (Sigma-Aldrich). After block, slides were exposed to primary antibody at 4 °C overnight, washed with PBS and incubated with Alexa Fluor donkey secondary antibodies (Invitrogen) for 1 h at room temperature. Antibodies used: chicken anti-GFP (Abcam, 13970), goat anti-OMP (Wako Chemicals, 544-10001), rabbit anti-GAP43 (Novus Biologicals, NB300-143). Images were acquired using a Zeiss LSM 880 confocal laser scanning microscope and processed using Zen3.3 (Zeiss) and Image J. Quantification of immunofluorescence was performed using ImageJ.

### In situ hybridization

In situ hybridization staining of decalcified sagittal skull section (described above) were performed using Advanced Cell Diagnostics (ACD) RNAscope system and probes. After rehydration of slides in PBS, slides were baked (30 min at 60 °C) and post-fixed in 4% PFA. Slides were dehydrated in progressive ethanol washes (50%, 70%, 100%, 100%, 5 min), air dried, treated with hydrogen peroxide (10 min). For in situ hybridization alone, Advanced Cell Diagnostics RNAscope 2.5 HD Detection Reagent—RED (322360) using standard manufactures protocol, RNAscope Target Retrieval Reagent (95–98 °C, 10 min), RNAscope Protease Plus (30 min), and standard hybridization with the Ldlrad3 probe (ACD,), signal amplification, and counter-staining with DAPI. For combined RNA–protein co-imaging, RNAscope Multiplex Fluorescent v2 Assay (323100) along with RNA–Protein Co-detection Ancillary Kit (323180) was used utilizing the Integrated Co-Detection Workflow (ICW). Following baking, post-fixation, dehydration, and hydrogen peroxide treatment, slides were immersed in Co-Detection Target Retrieval (95–98 °C, 5 min). Tissue was blocked and incubated overnight with GAP43 and OMP primary antibodies (see above) using Co-Detection Antibody Diluent. Samples were post-primary fixed using 10% neutral buffered formalin (30 min, RT) prior to RNAscope Protease Plus treatment, hybridization with the V-VEEV-ZPC-738 (ACD, 876381), Mm-Ldlrad3 (ACD, 872101), or dapB negative control probes (ACD, 310043), signal amplification with Opal 650 Dye (Akoya Biosciences, OP-001005) in RNAscope Multiplex TSA Buffer. Tissues were labeled with Alexa-conjugated secondary antibodies (see above) in Co-Detection Antibody Diluent, counter-stained with DAPI, and mounted in Prolong Gold (Invitrogen #P36930). Tissue were imaged as described above.

### Interferon treatment of mouse nasal mucosa

scRNAseq data set of intranasal IFNα mice was originally generated as described previously. Briefly, 8–10-week-old C57BL6/J mice received either 200 ng of IFNα (Biolegend 752802, ~ 1 × 10^4^ U) or saline intranasally (*N* = 2), Respiratory and olfactory mucosa were isolated 12 h later. Single cells suspension were generated in media containing Liberase (Roche) and DNase I (Roche) and loaded on duplicate Seq-Well S3 arrays for sequencing using Illumina NextSeq. Raw expression counts for cells previously defined within Immature Olfactory Sensory Neurons and Olfactory Sensory Neurons (saline and IFNα treated) clusters were downloaded from published data set. https://singlecell.broadinstitute.org/single_cell/study/SCP832?scpbr=the-alexandria-project#study-summary. Data were normalized and scaled using the Seurat R package (https://satijalab.org/seurat/). Differential expression tests between mature and immature OSNs within saline-treated group or between saline-treated or IFNα-treated OSNs were performed using Seurat FindAllMarkers function with default settings and Wilcoxon rank sum test (*P* value threshold = 0.05). GSEA analysis was performed using fgsea function from (fgsea, using the murine Gene Ontology gene sets (MSigDB). Genes were ordered by the Log2 fold change using Seurat FindMarkers function. Violin plots and heatmaps were generated using Seurat R package. Volcano plots were generated using the EnhancedVolcano package.

### Administration of IFNα

Recombinant mouse IFNα1 (Biolegend, 751806) was administered intranasally. Control mice were similarly administered vehicle solution of 0.1% bovine serum album (BSA) in PBS. Doses (8 × 10^4^ U, 10 µL/nostril) administered at time of infection were suspended in the inoculum under brief isoflurane anesthesia. Subsequent doses (8 × 10^4^ U, 5 µL/nostril) were administered at 1–3 h post-infection (hpi), as indicated, under brief isoflurane anesthesia.

### RNA isolation and quantitative RT-PCR

CNS and nasal cavity tissue was collected isolated from cardiac-perfused mice at various timepoints after intranasal ZPC-738 (10 pfu i.n.) infection and/or IFNα treatment. Total nasal cavity tissue, including the nasal turbinates, was collected using forceps following removal of the nasal bone along the nasomaxillary suture. RNA was isolated from tissues using RNeasy kit (Qiagen) according to manufacturer’s instructions, and quantified using a NanoDrop (Thermo Scientific). Following DNAse I treatment (Invitrogen) of RNA samples (1 µg) was reverse transcribed using Taqman Reverse Transcriptase kit (Applied Biosystems). qRT-PCR was performed using Power SYBR Green (Applied Biosystems) on a CFX384 PCR Detection System (Bio-Rad) using manufacturer’s recommended cycle parameters. Values are reported as the Cq values for target genes normalized to Cq values of GAPDH (Cq_gene_–Cq_GAPDH_). Primers (5′–3′) used are reported in Additional file 1: Table S1.

### Virologic analysis

At various post-infection intervals, nasal cavity and CNS tissue was collected from ZPC-738-infected mice after extensive cardiac perfusion with PBS. Viral titers were determined using standard plaque assay techniques by serial dilution of tissue homogenates over BHK cells, as described previously [18].

### Statistical analyses

Reported values are mean values ± standard error of the mean (SEM). Statistical analysis was performed using GraphPad Prism 7 software. Survival curves were analyzed by Mantel–Cox test. Cytokine and ISG expression in infected mice were analyzed via one-way analysis of variance (ANOVA), Bonferroni’s post hoc test was subsequently used for comparison of individual means. ISG expression following IFNα treatment were analyzed by unpaired Welch’s *t* test with Welch’s correction, as appropriate for samples with different variances. Weight loss and encephalitic sequelae scores were compared via two-way repeated measure ANOVA, followed by Bonferroni’s post hoc test. *P* values *P* < 0.05 were considered significant. Statistical values are indicated as follows *, *P* < 0.05; **, *P* < 0.01; ***, *P* < 0.001, ****, *P* < 0.0001 unless otherwise stated.

## Results

### Immature olfactory sensory neurons are the initial site of VEEV infection after intranasal exposure

**Fig. 1:**
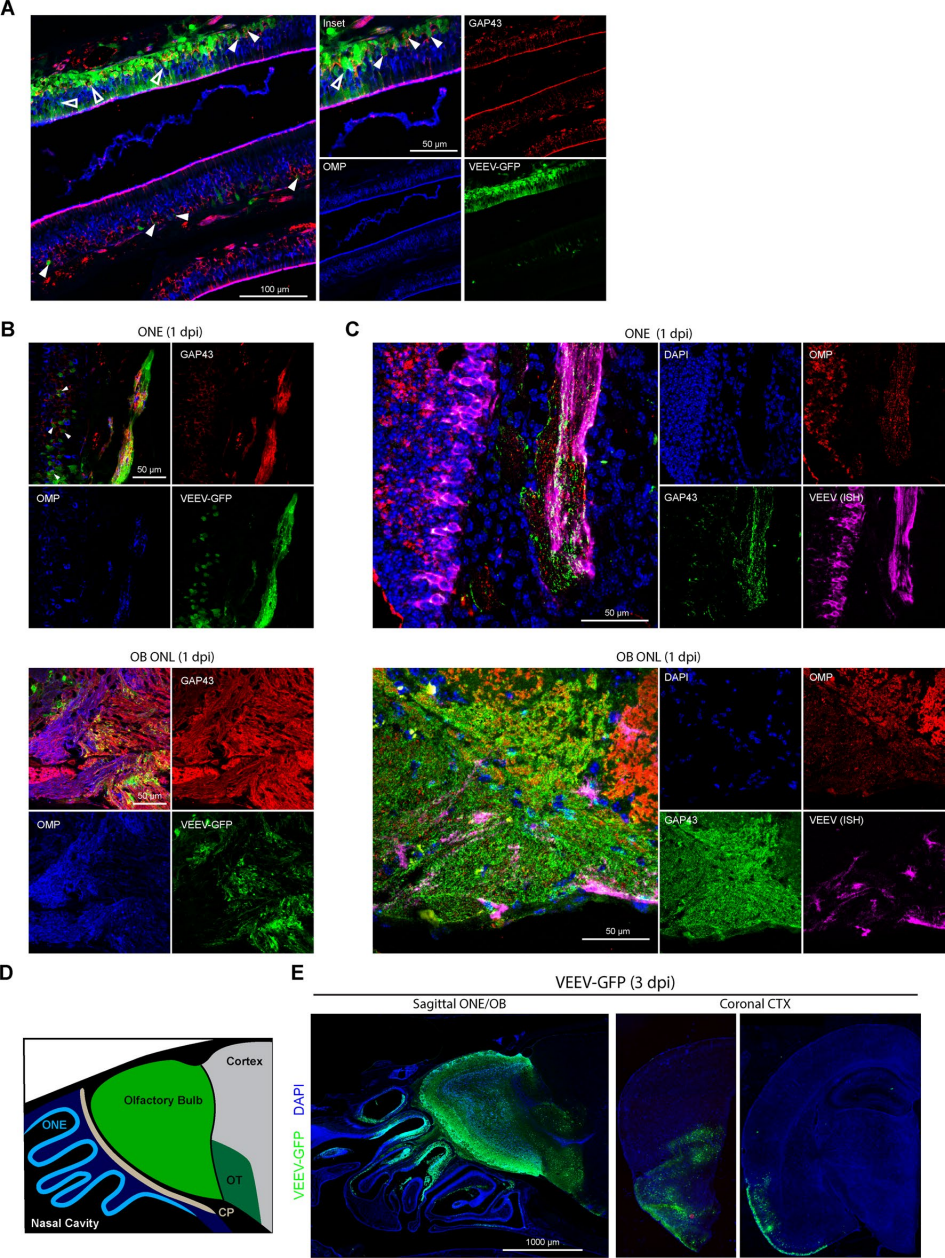
Neuroinvasion of intranasal VEEV involves early infection and anterograde transport from immature OSNs. **A**, **B** Immunostaining of murine ONE following intranasal VEEV–ZPC–GFP infection (50 pfu, 1 dpi). Solid and hollow arrowheads indicate GFP-labeling of infected GAP43+ immature OSNs (red) and OMP+ mature OSNs (blue), respectively. **B** GFP-labeling of OMP+ and GAP43+ OSN axons in the ONE and outer nerve layer of the olfactory bulb following ZPC–GFP infection (50 pfu, 1 dpi). **C** FlSH staining of VEEV genome (magenta) within the OMP+ (red) and GAP43+ (green) OSN axons in the ONE and outer nerve layer of the olfactory bulb (ZPC-738, 10 pfu, 1 dpi). **D** Cartoon depiction of sagittal section nasal cavity and forebrain. ONE (cyan), OB (green), olfactory tract (dark green) are indicated. **E** Immunostaining of intranasal VEEV–ZPC–GFP infection (50 pfu, 3 dpi) of sagittal sectioned nasal cavity and forebrain, and rostal sequence of coronal slices (Bregma ~ 2.0 and − 2.0 mm) depicting VEEV–GFP dissemination along lateral olfactory tract and piriform cortex. All images depict representative infections from *N* = 3 mice

Intranasal (i.n.) infection with an enzootic strain of VEEV (ID, ZPC-738; herein VEEV) results in rapid progression of weight loss and onset of encephalitic symptoms, with a mean survival time (MST) of 6.5 dpi (Additional file 2: Fig. S1A). In previous studies we demonstrated i.n. VEEV infection rapidly disseminates into the OB and CTX within 24 h, with viral loads peaking at 2–3 dpi in the OB and at 4–6 dpi in CTX, hindbrain regions, and spinal cord, highlighting early infection to the OB as a critical period to control VEEV dissemination along the olfactory route after i.n. exposure [3]. To define cellular tropism within the ONE we utilized a reporter strain of VEEV, ZPC-738-GFP, which exhibits similar virulence as the parent strain and labels infected cells green, in conjunction with OSN markers, all detected via double-label confocal microscopy [17]. Within the infected ONE, GAP43+ iOSNs are the earliest site of infection at 1 dpi [Fig. 1A, B (top) white arrowhead]. OMP+ mOSN were also infected at this timepoint [Fig. 1A (open arrowheads)]. GFP is also detected within GAP43+ and OMP+ OSN axons traversing the cribriform plate and within the olfactory nerve layer (ONL) of the OB (Fig. 1B, bottom). VEEV–RNA, as assessed via fluorescent in situ hybridization, is also detected within GAP43+ and OMP+ axons within the ONE and OB ONL (Fig. 1C, Additional file 2: Fig. S1B). By 3 dpi, GFP+ cells are observed throughout the ONE and OB, including the glomerular, mitral cell, and ganglion cell layers (Fig. 1D, E). At this timepoint, VEEV infection continues to spread into the forebrain along the olfactory tract and piriform CTX (Fig. 1E). Together, these data indicate that VEEV may utilize both immature and mature OSNs for anterograde transport into the OB.

### Higher levels of expression of a VEEV receptor, *Ldlrad3*, underlies enhanced tropism to iOSN

**Fig. 2:**
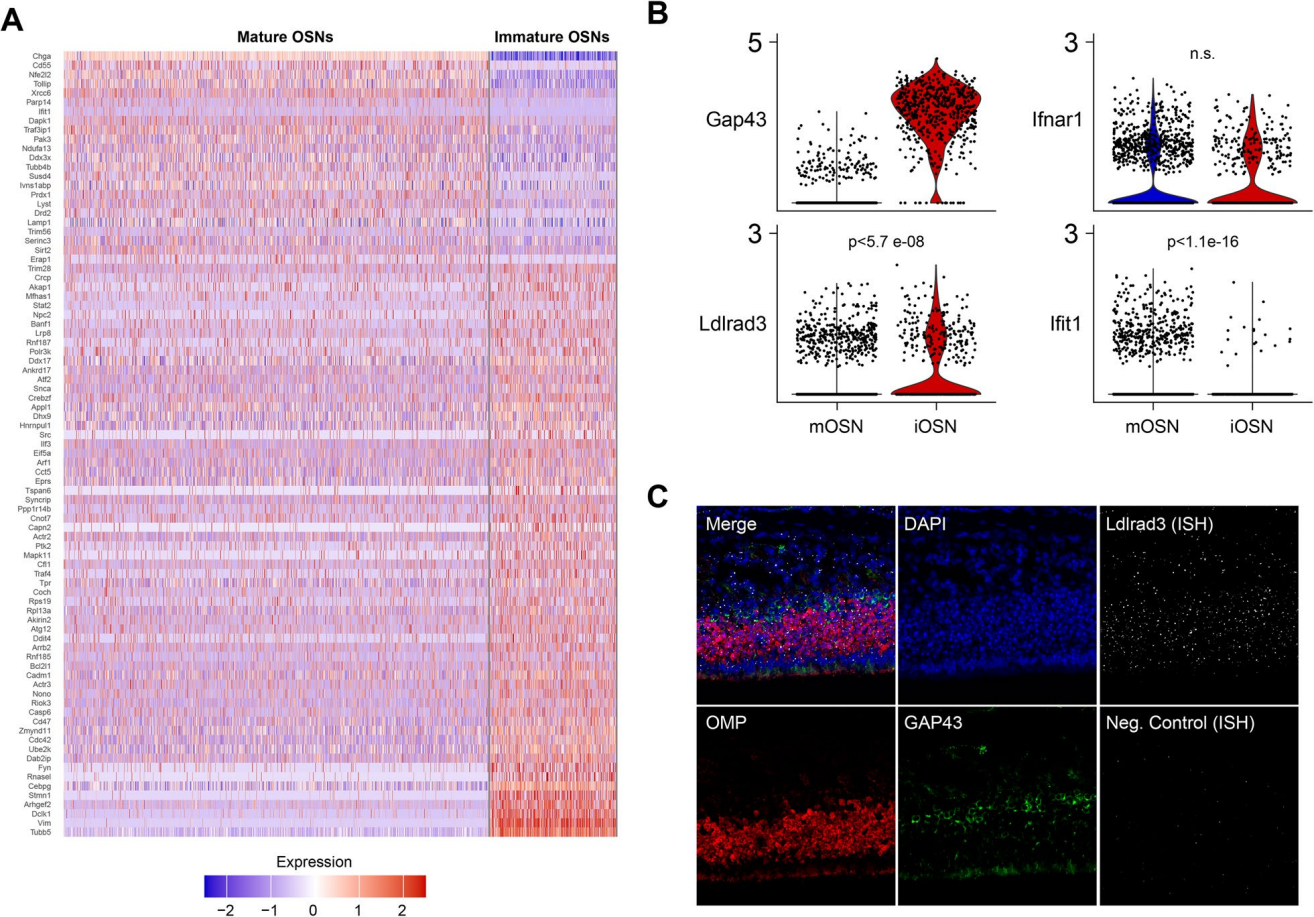
Differential expression of innate immune genes and LDLRAD3 in OSN. **A** Reanalysis of scRNA-seq data set (Ziegler et al., 2020) which captured a large population of both immature and mature murine OSN. Heatmap of DEGs belonging to GO terms (Innate Immune Response, Response to Virus, Response to Type-1 Interferon, Response to Interferon Alpha, and Response to Interferon Beta (*N* = 1816 mOSN, 539 iOSN from two mice). **B** Violin plot of expression of candidate genes relevant to VEEV infection and interferon signaling in mature and immature OSN. **C** FISH of Ldlrad3 (gray) expression in OMP+ (red) and GAP43+ (green) OSNs within the ONE. Images depict representative staining from *N* = 3 mice. *P* values are indicated

Despite the knowledge that peripheral neurons, including OSNs, are targets for many neurotropic viruses, there are few studies reporting their differential expression of viral entry receptors and innate immune responses [19–21]. To determine whether the SARS-CoV-2 entry receptor angiotensin converting enzyme (ACE2) is an ISG within cells of the ONE, Ziegler et al. performed single-cell RNA sequencing of murine nasal epithelium derived from mice 12 h after i.n. administration of saline versus IFNα (10^4^ U) [22]. While they found little to no ACE2 expression in iOSN or mOSN (with our without IFNα exposure), they provided a large data set for investigation of the differential expression of other viral entry receptor mRNAs and overall innate immune response networks in iOSN and mOSN [23]. To define differences between transcriptomic signatures of iOSN and mOSN that would underlie the observed enhanced infectivity of VEEV to iOSN, we analyzed differentially expressed genes (DEG) between the two cell types under saline treatment. As expected, the top DEG included genes involved in olfactory sensory perception, cilium development, and ion channel/transport protein expression (*Adcy3, Omp, Pde1c, Cngb1, Cnga4* (Additional file 3: Fig. S2A) [24, 25]. Similarly, gene set enrichment analysis (GSEA) using murine gene ontology (GO) pathways identified key differences in pathways associated with neuronal differentiation, axonal growth and synapse formation between iOSN and mOSN (Additional file 3: Fig. S2B). While mRNAs of genes relating to innate immunity or control of virus infection were not among the top DEG, some genes associated with GO pathways, including Innate Immune Response, Response to Virus, Response to Type 1 Interferon, Response to Interferon Alpha, and Response to Interferon Beta, were differentially expressed between iOSN and mOSN (Fig. 2A). For example, mRNA levels of Interferon Induced Protein with Tetratricopeptide Repeats 1 (*Ifit1*) was significantly higher in mOSN (Fig. 2B). However, none of these pathways were more significantly enriched by GSEA in either OSN population (Table 1), consistent with lack of differences in mRNA expression levels of IFNαβ receptor (*Ifnar1*) (Fig. 2B, Additional file 3: Fig. S2B). Together, broad differences in innate immunity do not explain the observed early VEEV tropism and infection of iOSN compared with mOSN. However, mRNA levels of a VEEV receptor, *Ldlrad3* [4], are significantly higher in iOSN versus mOSN (Fig. 2B). While detection of Ldlrad3 mRNA via fluorescent in situ hybridization (FISH) was observed in both iOSN and mOSN (Fig. 2C), it is likely that overall difference in levels of expression of *Ldlrad3* underlie earlier infection of iOSN.

**Table 1:** GSEA analysis of candidate innate immune GO pathways in mOSN and iOSN

GO pathway	ID	Adj. P Val	NES
Innate immune response	GO:0045087	0.68	1.081
Response to virus	GO:0009615	0.69	1.082
Response to type 1 interferon	GO:0034340	0.99	0.539
Response to interferon alpha	GO:0035455	0.94	− 0.683
Response to interferon beta	GO:0035456	0.80	0.99

### Intranasal IFNα administration induces rapid ISG expression in olfactory sensory neurons

**Fig. 3:**
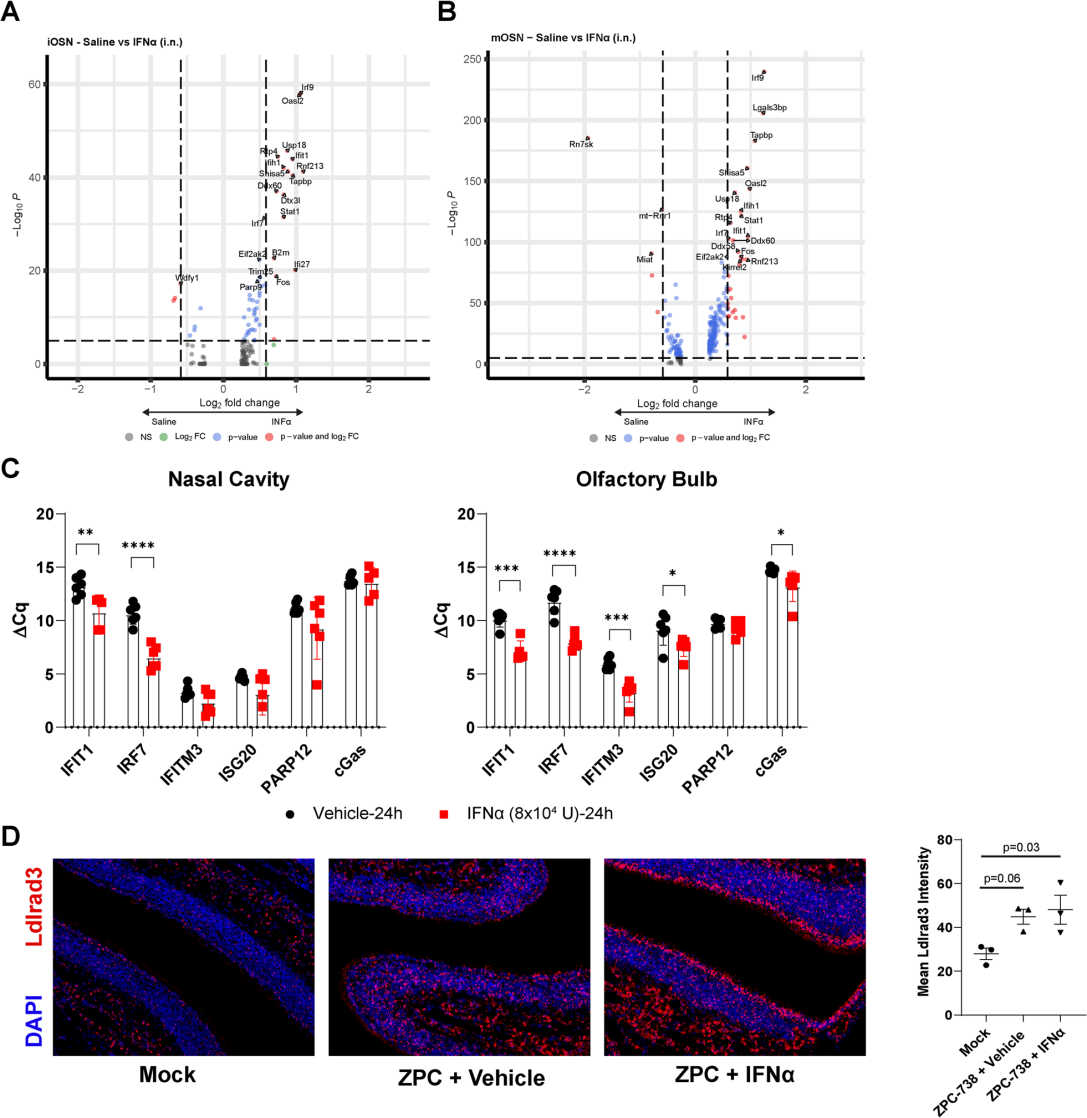
Single-dose intranasal IFNα treatment stimulates ISG expression in OSNs and OB. **A** Volcano plot of DEGs within immature OSNs following intranasal saline (negative) or IFNα (positive) treatment (1 × 10^4^ U, 12 h, *N* = 539 saline, 561 IFNα from two mice). **B** Volcano plot of DEGs within mature OSNs following intranasal saline (negative) or IFNα (positive) treatment (1 × 10^4^ U, 12 h, *N* = 1816 saline, 2076 IFNα from two mice). **C** Representative ISG expression within nasal cavity and olfactory bulb homogenates following intranasal IFNα (8 × 10^4^ U, 24 h, *N* = 5–6) treatment. **D** FISH of Ldlrad3 expression (red) following vehicle or IFNα co-administration during intranasal VEEV–ZPC (10 pfu, 12 hpi, *N* = 3). Error bars indicate mean ± SEM. ΔCq were compared via unpaired *t* test. Ldlrad3 expression was compared by one-way ANOVA, followed by Tukey multiple comparison. Statistical values are indicated as follows *P < 0.05; **P < 0.01; ***P < 0.001, ****P < 0.0001 unless otherwise stated

Given that OSN exhibit low levels of expression of innate immune molecules at baseline, we analyzed the scRNAseq data set deposited by Ziegler et al. for DEG in iOSN and mOSN following intranasal IFNα (10^4^ U,12 h) treatment [22, 23]. Both OSN cell types exhibit similar DEG (Fig. 3A, B). As expected DEGs and GSEA indicate strong enrichment of ISGs relating to Type 1 interferon responses following treatment, which was broader for mOSN (Additional file 3: Fig. S2C, D). Overall, this indicates that these cells, critical to early ONE replication and neuroinvasion into the OB, are responsive to such treatment. To validate ISG expression in the ONE and OB in a separate cohort of mice, we examined candidate ISG expression in total nasal cavity (NC) and OB following similar i.n. administration of recombinant murine IFNα (8 × 10^4^ U) followed by quantitative (q)PCR. Robust upregulation of ISG, including *Ifit1, Irf7, Ifitm3, Isg20, and cGas,* was observed in both the nasal cavity and OB at 24 h post-treatment with IFNα compared with vehicle-treated animals (Fig. 3C). To determine if IFNα also altered expression of Ldlrad3, we quantified *Ldlrad3* expression as assessed by FISH within the ONE following IFNα treatment at 12 hpi during VEEV infection (Fig. 3D, Additional file 2: Fig. S1C). While *Ldlrad3* expression was enhanced by VEEV infection, IFNα treatment did not synergistically impact its level of expression. Overall, these data indicate that IFNα treatment elicits rapid IFN response in both the ONE and OB, suggesting a potential therapeutic approach for limiting infection and neuroinvasion along this route.

### VEEV-mediated induction of endogenous IFN and ISGs is delayed within infected olfactory routes

**Fig. 4:**
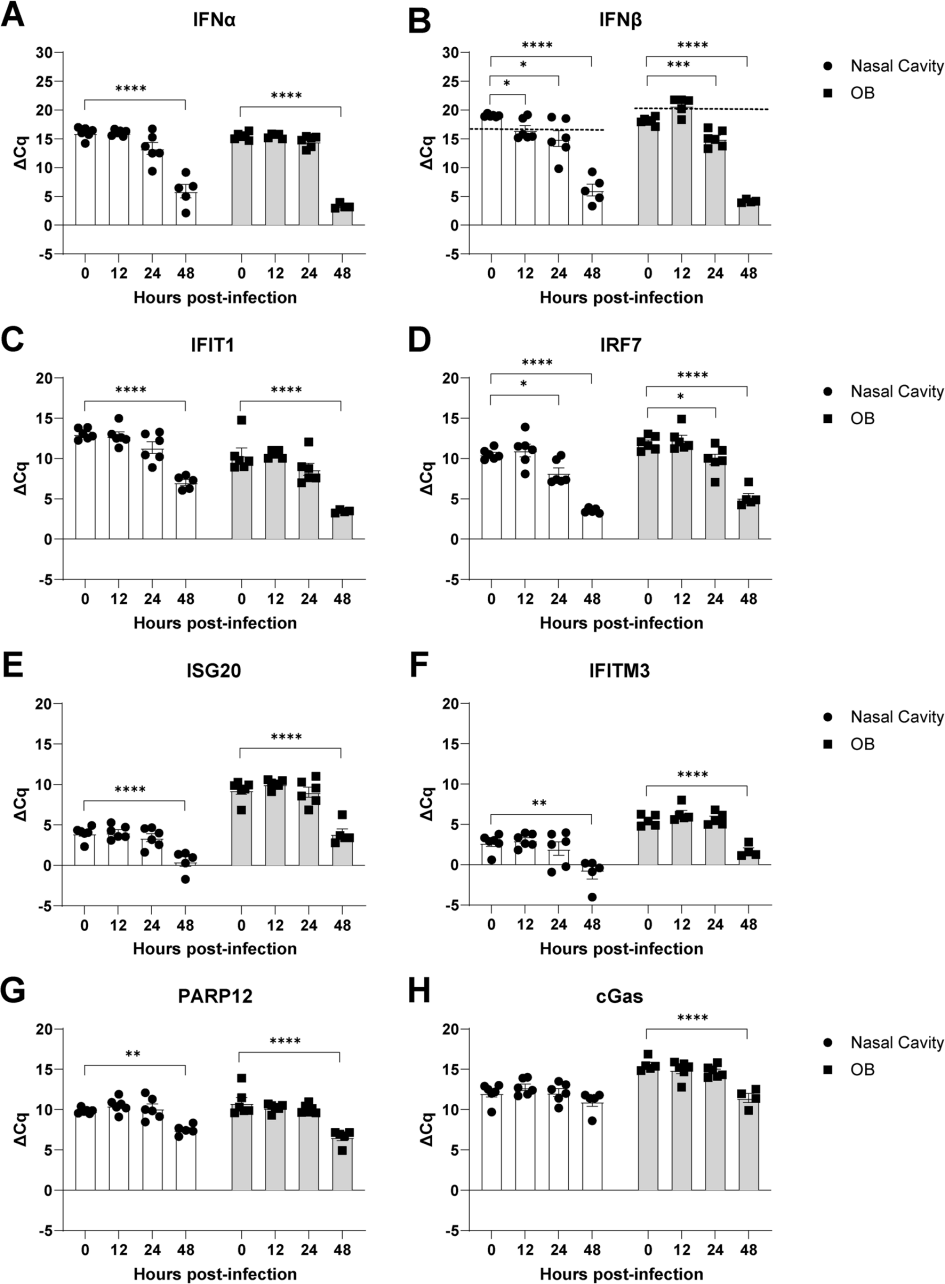
Interferon response in nasal cavity and OB is delayed relative to infection. **A**, **B** Induction of endogenous type-I interferons, IFNα (**A**) and IFNβ (**B**), expression within nasal cavity and olfactory bulb homogenates following intranasal inoculation of VEEV–ZPC-738 (10 pfu). **C**–**H** Upregulation of candidate interferon stimulated genes (ISGs) associated with restricting VEEV and/or alphavirus replication induced nasal cavity and olfactory bulb homogenates at various timepoints following intranasal inoculation of VEEV–ZPC-738 (10 pfu). Error bars indicate mean ± SEM, *N* = 5–6. ΔCq were compared via one-way ANOVA, followed by Dunnett’s multiple comparison test. Statistical values are indicated as follows *P < 0.05; **P < 0.01; ***P < 0.001, ****P < 0.0001 unless otherwise stated

While endogenous IFN signaling is critical for controlling VEEV infection in the periphery [7], the extent to which it controls VEEV infection and dissemination along the olfactory route is unknown. Knowledge of the kinetics of this response is also important for determining if exogenous administration of IFNα would be expected to limit VEEV neuroinvasion. To address this, IFN mRNA expression within the nasal cavity and OB was assessed in uninfected animals and at various timepoints (12, 24, 48 h) post-infection (hpi). IFNα mRNA is not significantly upregulated in the nasal cavity or OB until 48 hpi (Fig. 4A), while IFNβ mRNA induction is observed at 12 and 24 hpi within the nasal cavity, with significant induction at 24 hpi in the OB (Fig. 4B). Separate analyses of ISG mRNAs linked to inhibition of alphavirus infection showed similarly delayed onset of expression in the nasal cavity and OB (Fig. 4C–H) [26–31]. Only IRF7 was upregulated within 24 hpi (Fig. 4D), with all other candidates not exhibiting expression in both the NC and OB until 48 hpi (Fig. 4C–H). These data indicate potential windows of intervention with i.n. administered IFNα after i.n. exposure to VEEV.

### Intranasal IFNα treatment early after VEEV exposure delays morbidity and promotes survival

**Fig. 5:**
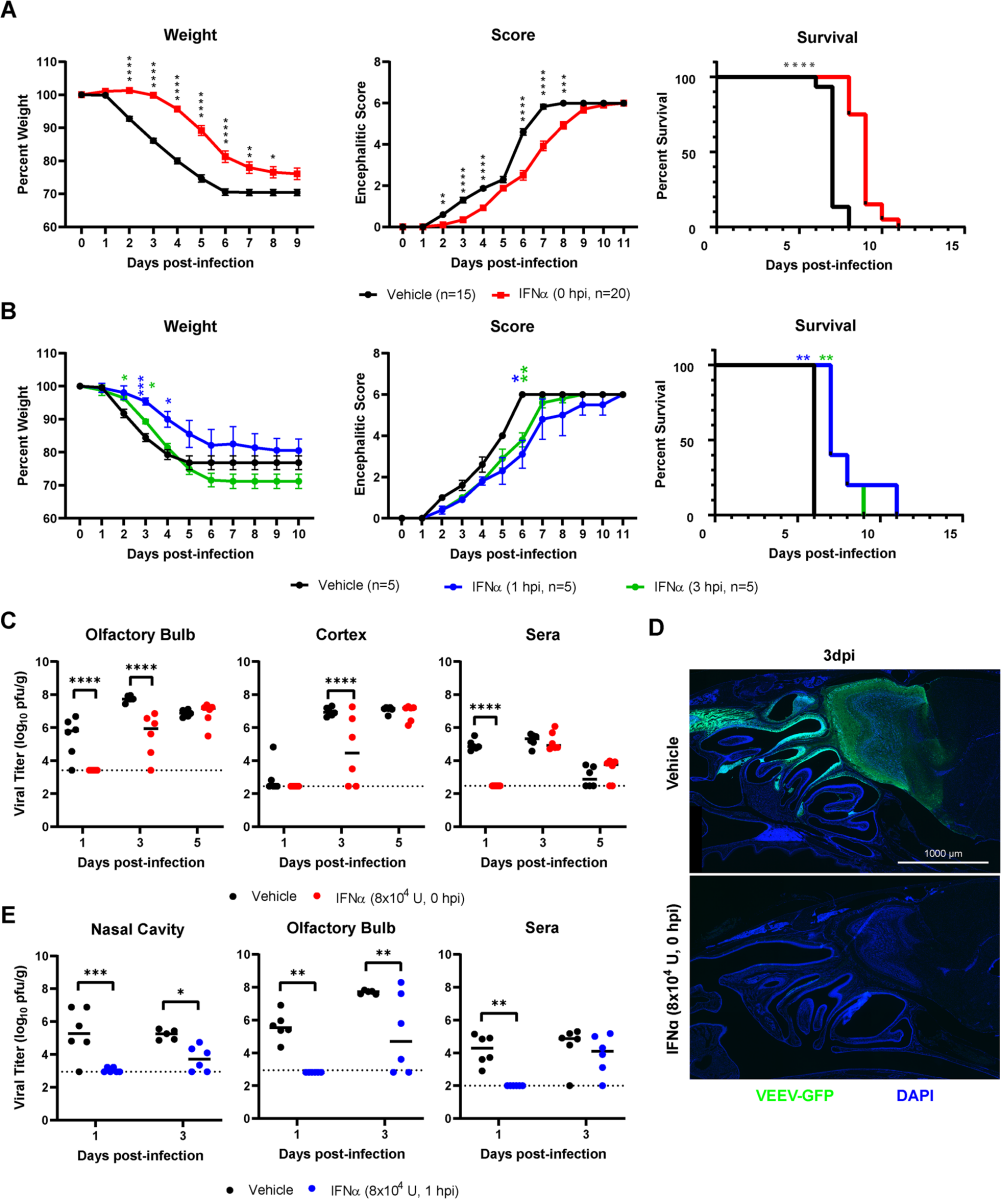
Intranasal IFNα delays morbidity and promotes survival during VEEV infection by suppressing onset of nasal and CNS infection. **A** Survival curves, weight loss curves, and encephalitis scores of mice co-administered single-dose intranasal IFNα during ZPC-738 (10 pfu) infection (*N* = 15–20 mice from two independent infections). **B** Survival curves, weight loss curves, and encephalitis scores of mice administered single-dose intranasal IFNα 1 or 3 h post-infection with VEEV–ZPC-738 (10 pfu) (*N* = 5). **C** Viral titers, as measured by plaque assay, from olfactory bulb, cortex, and sera collected at various DPI from mice co-administered intranasal IFNα during ZPC-738 (10 pfu) infection (*N* = 6). **D** Immunostaining of intranasal VEEV–ZPC–GFP infection (50 pfu, 3dpi) of sagittal sectioned nasal cavity and forebrain (representative example for *N* = 3 mice). **E** Viral titers from nasal cavity, olfactory bulb, and sera at various DPI to following intranasal IFNα administered 1 h following ZPC-738 (10 pfu) infection (*N* = 5–6). Weight loss, encephalitic sequelae scores, and titers were compared via two-way repeated measure ANOVA, followed by Bonferroni’s post hoc test. Survival curves were analyzed by Mantel–Cox test. Statistical values are indicated as follows *P < 0.05; **P < 0.01; ***P < 0.001, ****P < 0.0001 unless otherwise stated

To determine if i.n. administration of IFNα during early VEEV infection would improve outcomes following VEEV infection, IFNα treatment (8 × 10^4^ U) was administered concomitantly or at 1 and 3 h after i.n. VEEV infection (ZPC-738, 10 pfu) of wild-type mice. Pre-treatment (0 hpi) with IFNα delays morbidity, as assessed via encephalitic scoring and weight loss, compared with similarly infected vehicle-treated mice (Fig. 5A). Specifically, weight loss was significantly lower in IFNα-treated mice, and onset lagged approximately 2 days behind that of vehicle-treated VEEV-infected mice (Fig. 5A, left). While VEEV encephalitic signs were similar between vehicle- and IFNα-treated animals, scores were significantly lower and delayed by approximately 2 days in IFNα-treated mice (Fig. 5A, middle, Additional file 4: Fig. S3A). IFNα-treatment at the time of VEEV infection extended mean survival time (+ 2 dpi MST) but was ultimately insufficient to improve overall mortality (Fig. 5A, right). As knowledge of exposure to VEEV may be delayed, we determined whether post-infectious IFNα treatment at 1 or 3 hpi impacts disease and survival after i.n. infection with VEEV. Both treatment paradigms significantly delayed onset of encephalitic sequelae and weight loss compared with vehicle-treated VEEV-infected mice (Fig. 5B, left and middle, Additional file 4: Fig. S3B). However, weight was not as well-maintained as observed during concomitant IFNα and VEEV i.n. exposure compared with similarly infected vehicle-treated mice, especially when IFNα was administered at 3 hpi. Similar to concomitant treatment, IFNα treatment extended mean survival time (+ 2 dpi and + 1.6 dpi, respectively), without reducing overall mortality (Fig. 5B, right).

To determine if i.n. administration of IFNα limited VEEV replication within the ONE and/or CNS dissemination, viral titers were assessed in IFNα-treated animals. For mice administered IFNα at the time of VEEV infection (0 hpi), viral titers were assessed at 1, 3, and 5 dpi via standard plaque assays. In contrast with vehicle-treated animals, viral titers were undetectable in the brain (OB, CTX) or sera in IFNα-treated mice at 1 dpi (Fig. 5C). By 3 dpi, VEEV was detectable in the majority of CNS tissues derived from vehicle- and IFNα-treated mice (VEEV OB: 5/6; CTX: 4/6). However, overall VEEV titers in OBs and cortices derived from IFNα-treated mice were significantly reduced at this timepoint compared with similarly infected vehicle-treated animals (Fig. 5C). IFNα treatment, however, failed to control viremia beyond 1 dpi, as viral titers were equivalent in both treatment groups of VEEV-infected mice by 3 dpi. Similarly, VEEV viral titers in IFNα-treated mice reached equivalency to vehicle-treated mice by 5 dpi in all CNS regions (Fig. 5C). Direct observation of i.n. ZPC-738-GFP infection (50 pfu) in fixed whole skull mounts revealed strong suppression of infection in the ONE and OB at 3 dpi in IFNα-treated compared to vehicle-treated mice, which displayed robust ZPC-738-GFP throughout the ONE, OB, and olfactory tract (Fig. 5D). When infected cells were observed in IFNα-treated ONE, infected iOSNs were similarly observed earlier than mOSNs, suggesting that IFNα does not alter VEEV tropism beyond the period of suppression.

Endogenous IFNα and IFNβ expression correlated with the presence of VEEV during the time course of infection in the nasal cavity, OB, and cortices (Additional file 4: Fig. S3C, D). In IFNα-treated mice, endogenous IFNα and IFNβ expression was not as robustly induced until later in infection, correlating with the early suppression of VEEV in these mice. ISGs *Ifit1* and *IRF7*, which rapidly responded to i.n. IFNα previously, were induced by IFNα treatment in the nasal cavity and OB at 1 dpi, despite reduced endogenous Type I interferon expression (Additional file 4: Fig. S3E, F). Expression was similar, but significantly reduced, compared to vehicle control tissues through 3 dpi, suggesting that the effect of IFNα treatment on ISG expression may have begun to wane to levels that allow for VEEV replication during this period.

Finally, similar to concurrent (0 hpi) administration, i.n. IFNα treatment after infection (1 hpi) suppressed early infection, as VEEV viral titers at 1 dpi within the nasal cavity (NC), OB, and sera were undetectable compared with vehicle-treated mice (Fig. 5E). By 3 dpi, infection in the nasal cavity and OB remained significantly reduced in VEEV-infected mice treated with IFNα compared with vehicle-treated animals but was detectable in a majority of animals (VEEV+ NC: 4/6, VEEV+ OB: 4/6, VEEV+ Sera: 5/6).

## Discussion

In this study, we tracked the spread of VEEV infection from the ONE to the ONL of the OB, examining viral targets, innate immune responses, and the efficacy of post-exposure treatment with i.n. IFN. We found that GAP43+ iOSN were the first cells infected, followed by OMP+ mOSN, which both transport VEEV anterograde into the OB. scRNAreq analysis of OSN identified no broad innate immune deficits associated with iOSN to explain their enhanced infectivity compared to mOSN. However, specific changes in key ISGs, including significantly decreased levels of IFIT1 and/or increased expression of the VEEV receptor LDLRAD3 may underlie VEEV tropism to iOSN. The kinetics of ISG expression after i.n. VEEV revealed a significant delay, with robust upregulation occurring after 24 h. To determine if ISG levels could be rescued by exogenously administered IFN we utilized a model of i.n. IFNα treatment at the time of or post-exposure to VEEV infection. We found that IFNα treatment triggers early ISG expression in OSNs, the nasal cavity, and OB, even when administered as a single post-exposure dose. Consistent with this, IFNα treatment delayed onset of VEEV infection in the nasal cavity and OB, reduced encephalitic sequelae and extended survival. These data demonstrate that exogenous IFNα may be a potential post-exposure intervention for VEEV infection, allowing infected individuals time to obtain additional support or other treatments.

In concordance with our findings, previous studies have demonstrated VEEV infection of OSN; however, these studies did not distinguish tropism between iOSN versus mOSN [32, 33]. Axonal transport of VEEV has also been previously described, with detection of VEEV antigen and virions in olfactory nerve fibers crossing the cribriform plate [33]. Depending on their stage in maturation, iOSNs fully project to OB by ~ 7 days of differentiation, forming functional synapses in OB glomeruli that participate in limited olfaction [34–36]. As these neurons continue to express markers of immaturity, iOSNs represent not only an early site of VEEV infection within the ONE but also a route of anterograde transport to the OB. As VEEV infection propagates within the ONE, infected OMP+ mOSNs likely also contribute to additional VEEV anterograde transport of VEEV to OB but may be less critical to initial neuroinvasion along the olfactory tract.

Examination of genetic signatures of iOSN and mOSN at baseline and after IFNα exposure was performed via interrogation of a previously deposited scRNAseq data set [22, 23]. Type-1 IFN induces expression of ISGs, with only a few mediating the anti-viral activity for a specific pathogen. We found no broad deficits in innate immunity or antiviral gene expression at baseline to explain enhanced infectivity of iOSN over mOSN, with the exception of *Ifit1,* which was more highly expressed in mOSN. IFIT1 has been shown to limit VEEV replication by restricting translation of VEEV in strains that contain a G3A mutation, such as the TC-83 vaccine strain [27]. However, IFIT1 may be involved in other mechanisms that restrict VEEV replication, since within the same study, Ifit1^-/-^ mice exhibited shorter MST for both WT ZPC-738 and TC-83 (A3G) mutants. In addition, IFIT1 positively enhances ISG expression independently of viral RNA binding downstream of TLR4 activation in macrophages [37]. It is also possible that OSN differentiation induces other protective effects. Neuronal differentiation was observed to restrict VEEV infection in vitro using the AP7 olfactory-derived neuronal cell line [38]. This effect was cell intrinsic for differentiated cells and correlated with enhanced expression of interferon response factor (IRF)-3 and -7. Thus, additional screening of identified genes might be warranted. Most notably, mRNA expression of the VEEV receptor *Ldlrad3* was enhanced in iOSNs compared to mOSN. The endogenous ligand for LDLRAD3 is unknown, and is proposed to be distinct from other LDL receptor family members [39]. The role of LDLRAD3 in the maturation of iOSN is unknown; however, LDLRAD3 modulates amyloid precursor protein in neurons and promotes activity of E3 ubiquitin ligases, both of which impact neurogenesis [39–42].

Intranasal Type I IFN therapy has been explored for various respiratory viruses, including endemic viruses (rhinovirus and influenza) and recently SARS-CoV2 to modulate the severity of disease [43]. Similarly, Type I IFN treatment has been evaluated in other viruses considered to be potential biological weapons, including other encephalitic alphaviruses and hemorrhagic filoviruses, arenaviruses, phleboviruses [44–51]. However, similar studies evaluating the effectiveness intranasal IFN administration against intranasal/aerosol infection are limited [52, 53]. Our study demonstrates that the nasal cavity, including OSNs, responds rapidly to intranasal administration of IFN with detectable changes of ISG expression within 12 h. The antiviral state initiated following early IFN treatment after VEEV infection leads to suppression VEEV replication in the nasal cavity, preventing early expansion of VEEV infection and escape of VEEV into the blood. Previous studies have shown similar transient protection following intranasal VEEV infection, although these studies utilized prophylactic, multiday treatment and pegylation of IFNα (i.p.) [13]. However, IFN treatment is not able to control VEEV–CNS infection indefinitely. It remains unclear from which reservoir VEEV re-emerges after the effects of exogenous IFNα waned. It is possible that additional peripheral sites did not receive sufficient exogenous IFNα to fully prevent VEEV infection, allowing for infection of the ONE and OB via hematogenous routes. Alternatively, VEEV may eventually circumvent the induced IFN response within the ONE. This is also consistent with the delayed VEEV expansion in the ONE despite sustained expression of ISGs, such as *Ifit1 and Irf7,* throughout the course of VEEV infection. Encephalitic alphaviruses have evolved immune evasion mechanisms that inhibit host IFN responses and allow virus replication in infected cells. IFN signaling is suppressed by global shut-off of host transcription and translation and inhibition of STAT-1 signaling by capsid and capsid-independent mechanisms [8, 9, 12].

Intranasal delivery has been shown to enhance IFN delivery to rodent and non-human primate brain, especially the OB [14, 15]. Consistent with this model, we observed ISG expression in the OB of treated mice and sustained suppression of OB viral titers in the presence of normal viremia at 3 dpi. This may indicate additional protection of CNS infection downstream of ONE infection. We’ve reported previously that OB is also an early site of VEEV–CNS infection following subcutaneous infection [3]. Models utilizing subcutaneous or intravenous inoculation could potentially elucidate whether protection of OB is due to local IFN signaling or predominately secondary to delayed replication in the ONE.

Overall, intranasal IFN treatment delays onset of morbidity and extension of survival in a highly lethal animal model of VEEV infection. ZPC-738 is an enzootic strain that is completely lethal in mice. Such a disease course does not reflect the lethality associated with VEEV infection in humans. Approximately, < 1% of patients with VEEV succumb to the infection [1, 54]. Therefore, the IFN treatments strategies explored herein may yet be more effective in protecting against lethal VEEV encephalitis in patients. Certainly, early intervention will likely be the most effective, but additional studies evaluating sustained and late IFNα treatment are warranted. Repeated or chronic type I interferon therapy has been associated with side effects of flu-like symptoms, fatigue, weight-loss, and neurological sequalae, including cognitive impairment and depression [55]. These effects have been modeled in animals studies, and would need to be accounted for in repeated-treatment models [56–58]. However, sustained IFNα expressed by adenovirus vector has shown some promise of mitigating encephalitic alphavirus infection independently of high dose, bolus treatments [44, 46]. Alternatively, future studies may continue to leverage murine models to explore IFN modification and delivery strategies. Pegylation of Type I interferon sustains bioavailability, and improved outcomes against VEEV when administered i.p. [13]. Other modifications focusing on enhancing retention in the nasal cavity with modification or in situ mucoadhesive gel solution may be utilized [59, 60]. However, modifications would need to be evaluated for ease of delivery to the ONE, the effect on IFN delivery to CNS, and how VEEV neuroinvasion would be impacted, especially in those strategies that may disrupt the nose-to-brain barriers [61].

### Supplementary Information

**Additional file 1:** **Table S1.** QPCR Primers.

**Additional file 1: Fig S1.** Morbidity and survival curves for i.n. VEEV ZPC-738 infection. **A)** Model of intranasal inoculation of VEEV strain, ZPC-738 (10 pfu). Weight curves and encephalitis scores depict immediate and progressive weight loss and progression of morbidity of following infection. Survival curves depict lethality (6–7 DPI) of 8–10 week old C57BL/6J mice following ZPC-738 inoculation intranasal routes. **B)** Representative FlSH staining of VEEV genome (magenta) in naïve ONE counterstained with OMP + (red) and GAP43 + (green). **C)** Representative FISH staining of Ldlrad3 expression (red) or negative control probe in naive ONE. Error bars indicate mean ± SEM, *N* = 8 from two independent infections.

**Additional file 1:** **Fig S2.** Differential expression of OSN genes. **A)** Volcano plot of DEGs between mature (negative) and immature (positive) OSNs (*N* = 1816 mOSN, 539 iOSN from two mice). **B)** Top GO terms identified by GSEA analysis between mature (blue) and immature (red) OSNs ordered by normalized enrichment score (NES). **C)** Top GO terms of genes enriched genes as identified by GSEA analysis in iOSN (*N* = 539 saline, 561 IFNα from two independent mice) and mature OSNs (*N* = 1816 saline, 2076 IFNα from two mice) following intranasal saline (blue) or interferon alpha (red) treatment (1 × 10^4^ U, 12 h). **D)** Heatmap of DEGs belonging to GO terms (Innate Immune Response, Response to Virus, Response to Type-1 Interferon, Response to Interferon Alpha, and Response to Interferon Beta) between saline or IFNα treated mice.

**Additional file 1:** **Fig S3.** Intranasal IFNα treatment induces similar ISG response over duration VEEV infection despite delayed endogenous Type-1 interferon expression. **A)** Binned encephalitis scores of mice co-administered single-dose intranasal IFNα during ZPC-738 (10 pfu) infection (*N* = 15–20 mice from two independent infections). **B)** Binned encephalitis scores of mice administered single-dose intranasal IFNα 1 or 3 h post-infection with VEEV ZPC-738 (10 pfu) (*N* = 5). **C**, **D)** Induction of endogenous type-I interferons, IFNα **(C)** and IFNβ **(D)**, expression within nasal cavity, olfactory bulb, and cortex homogenates following intranasal IFNα treatment (8 × 10^4^ U) co-administered during VEEV-ZPC-738 (10 pfu) infection. **E**, **F)** Representative ISG expression within nasal cavity, olfactory bulb, and cortex homogenates following intranasal IFNα (8 × 10^4^ U, 0 hpi) treatment at multiple timepoints during ZPC-738 (10 pfu) infection (*N* = 5–6). Error bars indicate mean ± SEM, *N* = 8 from two independent infections. ΔCq were compared via unpaired t-test. Statistical values are indicated as follows *, *P* < 0.05; **, *P* < 0.01; ***, *P* < 0.001, ****, *P* < 0.0001 unless otherwise stated.

## Data Availability

The scRNAseq data set analyzed during the current study are available in the Broad Institute repository, https://singlecell.broadinstitute.org/single_cell/study/SCP832?scpbr=the-alexandria-project#study-summary. All other data generated and analyzed during this study are included in the published article and its supplementary information files.

## Abbreviations

CHIKV: Chikungunya virus
SINV: Sindbis virus
SFV: Semliki Forest virus
RRV: Ross River virus
VEEV, EEEV, WEEV: Venezuelan, Eastern, and Western equine encephalitis viruses
i.n.: Intranasal
OSN: Olfactory sensory neuron
LDLRAD3: Low Density Lipoprotein Receptor Class A Domain Containing 3
IFN: Type I interferons
ISG: Interferon-stimulated genes
OB: Olfactory bulb
CTX: Cortex
ONE: Olfactory neuroepithelium
ONL: Olfactory nerve layer
ACE2: Angiotensin converting enzyme 2
GSEA: Gene set enrichment analysis
GO: Gene ontology
IFIT1: Interferon Induced Protein with Tetratricopeptide Repeats 1
DEG: Differentially expressed genes
scRNAseq: Single cell RNA sequencing
IRF: Interferon response factor
