# Supplementary material for: Post-exposure intranasal IFNα suppresses replication and neuroinvasion of Venezuelan Equine Encephalitis virus within olfactory sensory neurons
Source: J Neuroinflammation. 2024 Jan 17;21:24. doi: 10.1186/s12974-023-02960-1 (PMC10792865; doi:10.1186/s12974-023-02960-1)
Supplement: Supplementary file 1 — Additional file 1: Table S1. QPCR Primers. [file 12974_2023_2960_MOESM1_ESM.docx]

**Table S1: QPCR Primers**

| **Gene** | **Fwd Primer** | **Rev Primer** |
| --- | --- | --- |
| **GAPDH** | GGCAAATTCAACGGCACAGT | AGATGGTGATGGGCTTCCC |
| **IFNα** | CTTCCACAGGATCACTGTGTACCT | TTCTGCTCTGACCACCTCCC |
| **IFNβ** | CTGGAGCAGCTGAATGGAAAG | CTTCTCCGTCATCTCCATAGGG |
| **IFIT1** | GAACCCATTGGGGATGCACAACCT | CTTGTCCAGGTAGATCTGGGCTTCT |
| **IRF7** | ATTTCGGTCGTAGGGATCTG | GTTGGTCTTCCAGCCTCTTC |
| **IFITM3** | GCCTATGCCTACTCCGTGAAGT | GCCTGGGCTCCAGTCACAT |
| **ISG20** | AACATCCAGAACAACTGGCGG | GTCTGACGTCCCAGGGCA |
| **PARP12** | AGACCGGGAAGAACTGTAGGA | TTTGGAAGGAGCAAGAGCCG |
| **cGas** | ACGAGAGCCGTTTTATCTCGTACCC | TGTCCGGAAGATTCACAGCATGTTT |
