# Supplementary material for: Post-exposure intranasal IFNα suppresses replication and neuroinvasion of Venezuelan Equine Encephalitis virus within olfactory sensory neurons
Source: J Neuroinflammation. 2024 Jan 17;21:24. doi: 10.1186/s12974-023-02960-1 (PMC10792865; doi:10.1186/s12974-023-02960-1)

**Fig S1. Morbidity and survival curves for i.n. VEEV ZPC-738 infection.**

**A)** Model of intranasal inoculation of VEEV strain, ZPC-738, at increasing doses (10 pfu). Weight curves and encephalitis scores depict immediate and progressive weight loss and progression of morbidity of following infection. Survival curves depict lethality (6-7 DPI) of 8-10 week old C57BL/6J mice following ZPC-738 inoculation intranasal routes.  **B)** Representative FlSH staining of VEEV genome (magenta) in naïve ONE counterstained with OMP+ (red) and GAP43+ (green). **C)** Representative FISH staining of Ldlrad3 expression (red) or negative control probe in naive ONE. Error bars indicate mean ± SEM, N=8 from two independent infections.


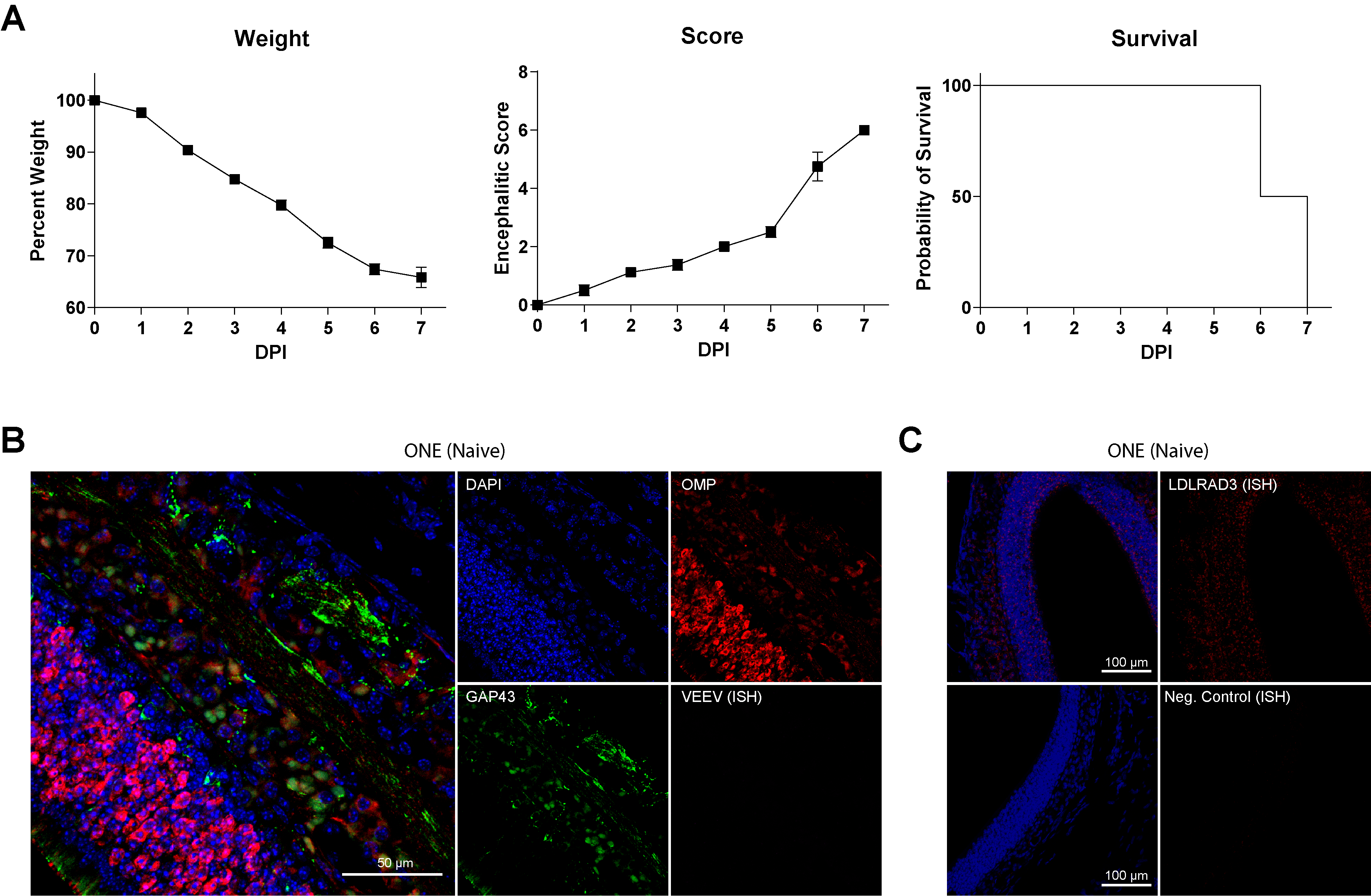

Supplement: Supplementary file 2 — Additional file 1: Fig S1. Morbidity and survival curves for i.n. VEEV ZPC-738 infection. A) Model of intranasal inoculation of VEEV strain, ZPC-738 (10 pfu). Weight curves and encephalitis scores depict immediate and progressive weight loss and progression of morbidity of following infection. Survival curves depict lethality (6–7 DPI) of 8–10 week old C57BL/6J mice following ZPC-738 inoculation intranasal routes. B) Representative FlSH staining of VEEV genome (magenta) in naïve ONE counterstained with OMP + (red) and GAP43 + (green). C) Representative FISH staining of Ldlrad3 expression (red) or negative control probe in naive ONE. Error bars indicate mean ± SEM, N = 8 from two independent infections. [file 12974_2023_2960_MOESM2_ESM.docx]
