# Supplementary material for: Post-exposure intranasal IFNα suppresses replication and neuroinvasion of Venezuelan Equine Encephalitis virus within olfactory sensory neurons
Source: J Neuroinflammation. 2024 Jan 17;21:24. doi: 10.1186/s12974-023-02960-1 (PMC10792865; doi:10.1186/s12974-023-02960-1)

**Fig** **S2.** **Differential expression of OSN genes**

**A)**  Volcano plot of DEGs between mature (negative) and immature (positive) OSNs (N=1816 mOSN, 539 iOSN from two mice). **B)** Top GO terms identified by GSEA analysis between mature (blue) and immature (red) OSNs ordered by normalized enrichment score (NES). **C)** Top GO terms of genes enriched genes as identified by GSEA analysis in iOSN (N=539 saline, 561 IFNα from two independent mice) and mature OSNs (N= 1816 saline, 2076 IFNα from two mice) following intranasal saline (blue) or interferon alpha (red) treatment (1x10^4^ U, 12 hours).  **D)** Heatmap of DEGs belonging to GO terms (Innate Immune Response, Response to Virus, Response to Type-1 Interferon, Response to Interferon Alpha, and Response to Interferon Beta between saline or IFNα treated mice.


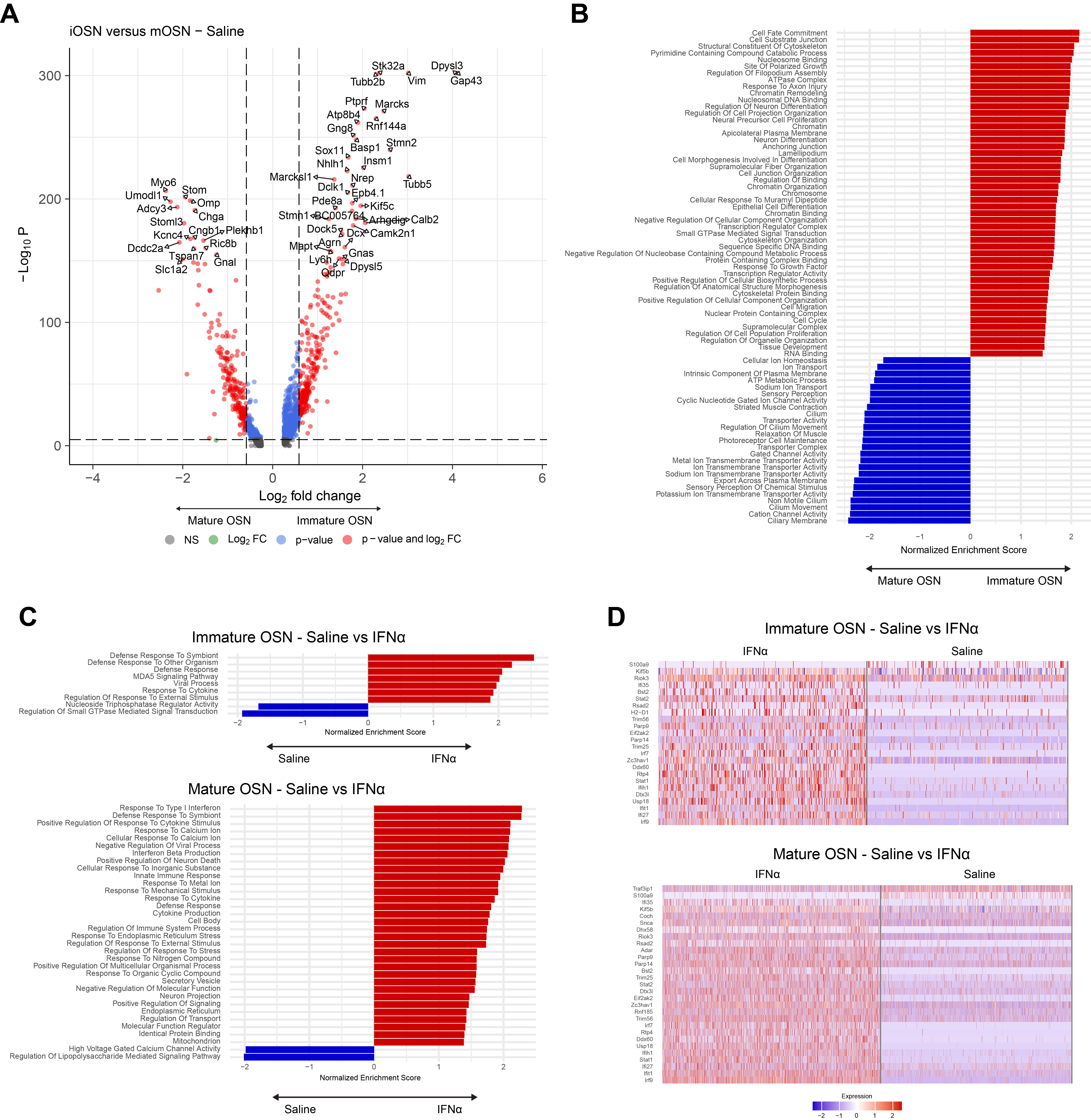

Supplement: Supplementary file 3 — Additional file 1: Fig S2. Differential expression of OSN genes. A) Volcano plot of DEGs between mature (negative) and immature (positive) OSNs (N = 1816 mOSN, 539 iOSN from two mice). B) Top GO terms identified by GSEA analysis between mature (blue) and immature (red) OSNs ordered by normalized enrichment score (NES). C) Top GO terms of genes enriched genes as identified by GSEA analysis in iOSN (N = 539 saline, 561 IFNα from two independent mice) and mature OSNs (N = 1816 saline, 2076 IFNα from two mice) following intranasal saline (blue) or interferon alpha (red) treatment (1 × 10^4^ U, 12 h). D) Heatmap of DEGs belonging to GO terms (Innate Immune Response, Response to Virus, Response to Type-1 Interferon, Response to Interferon Alpha, and Response to Interferon Beta) between saline or IFNα treated mice. [file 12974_2023_2960_MOESM3_ESM.docx]
