# Supplementary material for: Post-exposure intranasal IFNα suppresses replication and neuroinvasion of Venezuelan Equine Encephalitis virus within olfactory sensory neurons
Source: J Neuroinflammation. 2024 Jan 17;21:24. doi: 10.1186/s12974-023-02960-1 (PMC10792865; doi:10.1186/s12974-023-02960-1)

**Fig S3. Intranasal IFNα treatment induces similar ISG response over duration VEEV infection despite delayed endogenous Type-1 interferon expression.**

**A)** Binned encephalitis scores of mice co-administered single-dose intranasal IFNα during ZPC-738 (10 pfu) infection (N=15-20 mice from two independent infections).  **B)** Binned encephalitis scores of mice administered single-dose intranasal IFNα 1 or 3 hour post-infection with VEEV ZPC-738 (10 pfu) (N=5). **-C-D)** Induction of endogenous type-I interferons, IFNα **(C)** and IFNβ **(D)**, expression within nasal cavity, olfactory bulb, and cortex homogenates following intranasal IFNα treatment (8x10^4^ U) co-administered during VEEV-ZPC-738 (10 pfu) infection. **E-F)** Representative ISG expression within nasal cavity, olfactory bulb, and cortex homogenates following intranasal IFNα (8x10^4^ U, 0 hpi) treatment at multiple timepoints during ZPC-738 (10 pfu) infection (N=5-6). Error bars indicate mean ± SEM, N=8 from two independent infections.

ΔCq were compared via unpaired t-test. Statistical values are indicated as follows *, *P*<0.05; **, *P*<0.01; ***, *P*<0.001, ****, P<0.0001 unless otherwise stated.


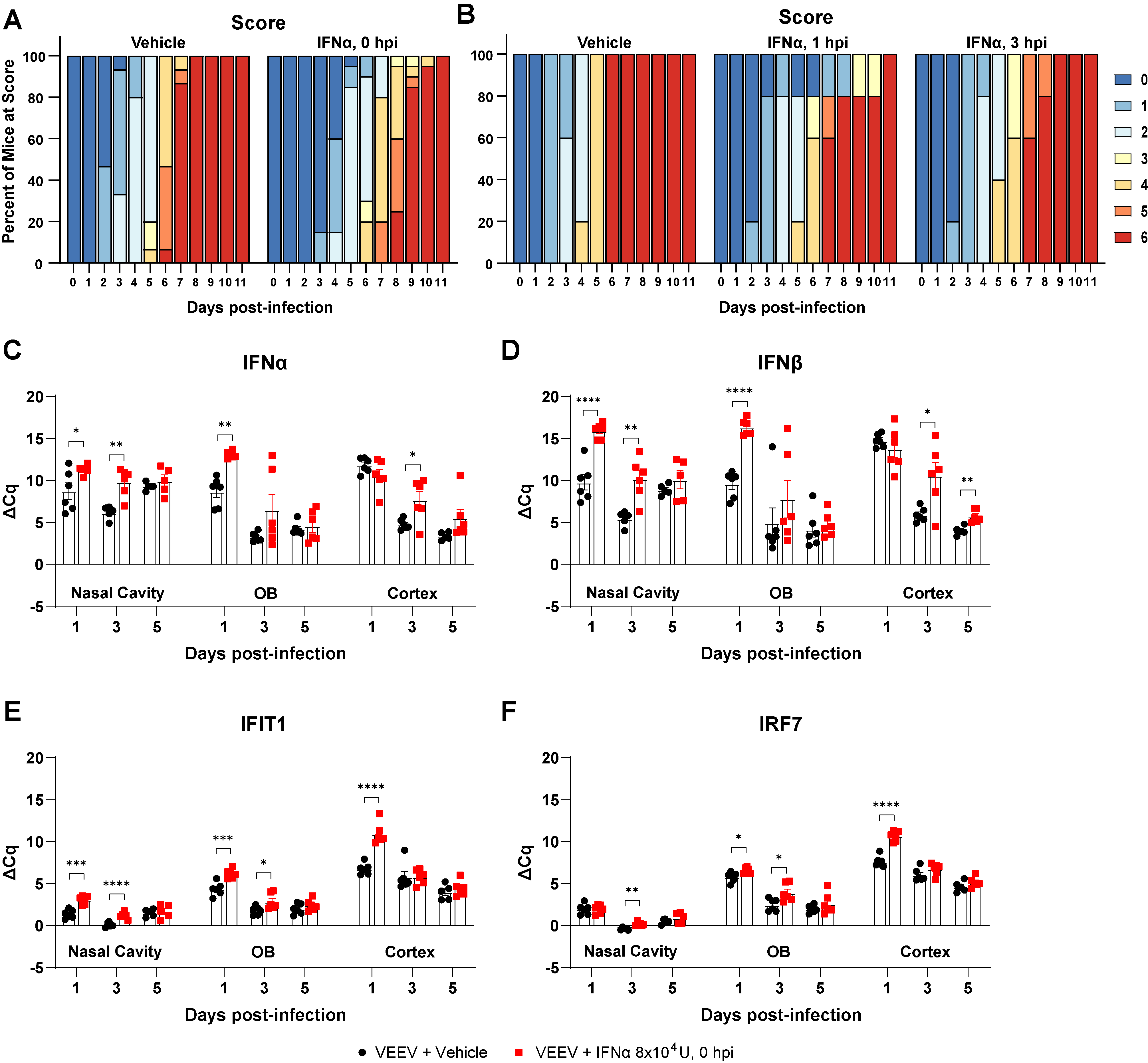

Supplement: Supplementary file 4 — Additional file 1: Fig S3. Intranasal IFNα treatment induces similar ISG response over duration VEEV infection despite delayed endogenous Type-1 interferon expression. A) Binned encephalitis scores of mice co-administered single-dose intranasal IFNα during ZPC-738 (10 pfu) infection (N = 15–20 mice from two independent infections). B) Binned encephalitis scores of mice administered single-dose intranasal IFNα 1 or 3 h post-infection with VEEV ZPC-738 (10 pfu) (N = 5). C, D) Induction of endogenous type-I interferons, IFNα (C) and IFNβ (D), expression within nasal cavity, olfactory bulb, and cortex homogenates following intranasal IFNα treatment (8 × 10^4^ U) co-administered during VEEV-ZPC-738 (10 pfu) infection. E, F) Representative ISG expression within nasal cavity, olfactory bulb, and cortex homogenates following intranasal IFNα (8 × 10^4^ U, 0 hpi) treatment at multiple timepoints during ZPC-738 (10 pfu) infection (N = 5–6). Error bars indicate mean ± SEM, N = 8 from two independent infections. ΔCq were compared via unpaired t-test. Statistical values are indicated as follows *, P < 0.05; **, P < 0.01; ***, P < 0.001, ****, P < 0.0001 unless otherwise stated. [file 12974_2023_2960_MOESM4_ESM.docx]
